# Characterization of intestinal fungal community diversity in people living with HIV/AIDS (PLWHA)

**DOI:** 10.1186/s12981-023-00589-x

**Published:** 2024-02-13

**Authors:** Pengfei Meng, Guichun Zhang, Xiuxia Ma, Xue Ding, Xiyuan Song, Shuyuan Dang, Ruihan Yang, Liran Xu

**Affiliations:** 1 Henan University of Chinese Medicine, Zhengzhou, 450000 China; 2 The First Affiliated Hospital of Henan University of Chinese Medicine, Zhengzhou, 450000 China

**Keywords:** HIV, AIDS, Intestinal fungi, ITS, Inflammatory factors

## Abstract

Acquired Immune Deficiency Syndrome (AIDS) is a highly dangerous infectious disease caused by the Human Immunodeficiency Virus (HIV), a virus that attacks the human immune system. To explore the correlation between intestinal fungal community and immune function (Immune cells and inflammatory factors) in people living with HIV/AIDS (PLWHA). The feces and blood samples were collected from two groups of subjects: PLWHA and healthy controls. High-throughput sequencing of the internal transcribed spacer 1, flow cytometry, and ELISA were performed to analyze the differences and correlations between fungal microbiota, cellular immune status and serum inflammatory factors in the two groups. There were significant differences in the composition of fungal microbiota between the two groups. The relative abundance of *Candida*, *Bjerkandera*, and *Xeromyces* in PLWHA was significantly higher than that of healthy volunteers (*P* < 0.01), while the relative abundance of *Mycospaerella*, *Xeroxysium*, *Penicillium*, and *Glomerella* in PLWHA was significantly lower than that of healthy volunteers. The correlation analysis results show that *Mycospaerella* and *Xeromyces* are significantly positively correlated with CD4^+^/CD8^+^ T cells and the anti-inflammatory cytokine IL-4. On the other hand, *Candida* was positively correlated with pro-inflammatory factors negatively correlated with CD4^+^/CD8^+^ T cells and the anti-inflammatory cytokine IL-4, while it is positively correlated with pro-inflammatory cytokines. The significant increase in the relative abundance of *Candida* may be one of the important causes of intestinal damage in PLWHA. The results of this study contribute to the understanding of the relationship between fungal microbiota structure and immune function in the gut ecology of PLWHA.

**Supplementary Information:**

The online version contains supplementary material available at 10.1186/s12981-023-00589-x.

## Introduction

The natural course of HIV-1 infection is mainly characterized by persistent virus replication, progressive decline in CD4^+^ T cell count, ultimately leading to severe immune deficiency in the human body, causing various opportunistic infections, tumors, and even death [1–3]. The gastrointestinal T cells are the primary target of HIV [4]. HIV-related diarrhea is one of the most common complications in the middle and late stages of AIDS [5]. The main pathological site is the intestine, which is a digestive symptom caused by damage to the intestinal mucosa, impaired intestinal barrier function (increased intestinal permeability) and impaired immune function [6, 7]. The main clinical symptoms include diarrhea, malnutrition, and weight loss, which is one of the main causes of decreased quality of life and even death in patients [8].

The human intestinal tract harbors a large number of microorganisms, and the dynamic balance of these microorganisms is closely related to normal human life activities [9]. Dysbiosis of the gut microbiota can lead to the occurrence of various diseases and promote disease progression [10, 11]. After the HIV invades the human body, it targets CD4^+^ T cells and disrupts the immune system by replicating extensively and changing the permeability of cell membranes, thereby increasing the likelihood of various opportunistic infections [12]. The gastrointestinal tract is the primary site of residence for CD4^+^ T lymphocytes [13]. Their destruction leads to damage to the intestinal mucosal barrier, allowing the overgrowth and displacement of gut microbiota, which can trigger other inflammation-related complications and worsen infections, ultimately leading to death [14]. In addition, since the intestinal mucosal barrier is not repaired, bacterial translocation and infection rates do not decrease [15]. Therefore, current AIDS treatment may also be achieved through in-depth research on changes in the gut microbiota. Studies have found that probiotics can significantly improve gut microbiota dysbiosis in PLWHA, especially those not undergoing HAART treatment [16]. Some researchers have compared the gut microbial communities of children living with HIV/AIDS (CLWHA) and healthy children and found that HAART can help restore or approximate the abundance of gut microbiota in CLWHA to that of healthy individuals [17, 18].

Intestinal mucosal damage is the core of the pathogenesis of AIDS, and the ectopic gut microbiota and its metabolites are key drivers of intestinal mucosal damage [19, 20]. Current research on the gut microbiota of PLWHA mostly focuses on bacterial communities and their metabolites [21–23]. Although the proportion of fungal communities in the human microbiome is less than 1%, fungal communities play a crucial role in maintaining microbiome structure balance, stable metabolic function, and host immune response [24]. When the human immune system is damaged or the normal microbial community is dysregulated, the antagonistic effect of intestinal bacteria against fungi will weaken, and the balance between microorganisms will be disrupted, exerting negative effects on the host. The most common *Candida* in the human intestine can transition from a non-pathogenic symbiotic state to a pathogenic state [25]. The detection rate or infection rate of *Candida* is high in the oral mucosa, urogenital tract, and other areas that are connected to the outside world in PLWHA. Research has shown that damage to the physical structure of the intestinal mucosa, imbalance of gut microbiota, and decreased host immune function can all promote fungal infection [26]. In addition, it has shown that damage to the physical structure of the intestinal mucosa, imbalance of gut microbiota and decrease host immune function can all promote fungal infection. This indicated that PLWHA are at risk of conditionally pathogenic fungal infections [26]. Therefore, studying fungal communities in the gut microbiota is important for understanding the pathogenesis of AIDS and delaying disease progression. As a consequence, this study characterized the differences in gut fungal communities, immune function, and inflammatory factors between PLWHA and healthy individuals as well as their correlations through high-throughput sequencing technology. For the subsequent development of precise interventions targeting intestinal fungal community disorders, including correcting intestinal fungal community disorders by introducing exogenous microorganisms, or inhibiting the damage of related fungal to the intestinal mucosal barrier, providing a basis for improving gastrointestinal symptoms in PLWHA.

## Materials and methods

### Case selection

After inclusion and exclusion criteria (Supplementary Material S1), a total of 41 PLWHA who visited the First Affiliated Hospital of Henan University of Traditional Chinese Medicine from May 2018 to April 2020, as well as 12 healthy volunteers, were included in the study (Clinical data is shown in Table 1). In addition, prior to the start of this study, informed consent was obtained from the study subjects, and the research protocol was approved by the Ethics Committee of the First Affiliated Hospital of Henan University of Traditional Chinese Medicine (2018HL-044-01 and 2019HL-099-01).

**Table 1:** Clinical data of each group

Clinical data	CON (n = 12)	HIV (n = 41)	*P*-value
**Age (year)**	51.17 ± 7.38	47.65 ± 12.17	0.308
**Gender (Male/Female)**	7/5	28/13	0.156
**Diet**			
**Coarse cereals**			0.261
^a^Frequent	1 (8.3%)	4 (9.8%)	
^b^Occasional	9 (75.0%)	31 (75.6%)	
^c^Rare	2 (16.7%)	6 (14.6%)	
**Vegetable**			0.541
Frequent	4 (33.3%)	10 (24.4%)	
Occasional	8 (66.7%)	28 (68.3%)	
Rare	0 (0.0%)	3 (7.3%)	
**Fruit**			0.639
Frequent	5 (41.7%)	16 (39.0%)	
Occasional	7 (58.3%)	22 (53.7%)	
Rare	0 (0.0%)	3 (7.3%)	
**Oily foods**			0.367
Frequent	1 (8.3%)	2 (4.9%)	
Occasional	8 (66.7%)	31 (75.6%)	
Rare	3 (25.0%)	8 (19.5%)	
**BMI (kg/m** ^**2**^ **)**	24.5 ± 3.2	22.1 ± 2.5	0.145
**Smoking**	2 (16.7%)	5 (12.2%)	0.652
**Drinking**	1 (8.3%)	2 (4.9%)	0.569
**Transmission route**	Not Applicable		Not Applicable
Blood transmission		20 (48.8%)	
Heterosexual behavior		15 (43.9%)	
Homosexual behavior		2 (4.9%)	
Intravenous drug injection		1 (2.4%)	
**HAART scheme**	Not Applicable		Not Applicable
^d^3TC+TDF + EFV		28 (68.3%)	
^e^EVG/c/FTC/TAF		8 (19.5%)	
^f^Zidovudine and Lamivudine Tablets + LPV/r		5 (12.2%)	

*Note:* ^a^ Frequent indicated ≥ 5 times per week

^b^ Occasional indicated 2–4 times per week

^c^ Rare indicated ≤ 1 time per week

^d^ 3TC + TDF + EFV refers to a combination therapy of three drugs, Efavirz, Lamivudine and Tenofovir

^e^ EVG/c/FTC/TAF refers to a combination of four drugs, Elvitegravir, Cobicistat, Emtricitabine and Tenofovir alafenamide

^f^ Zidovudine tablets are a drug combination of Lamivudine and Zidovudine. LPV/r is a drug combination of Lopinavir and Ritonavir

### Sample collection

**Fig. 1:**
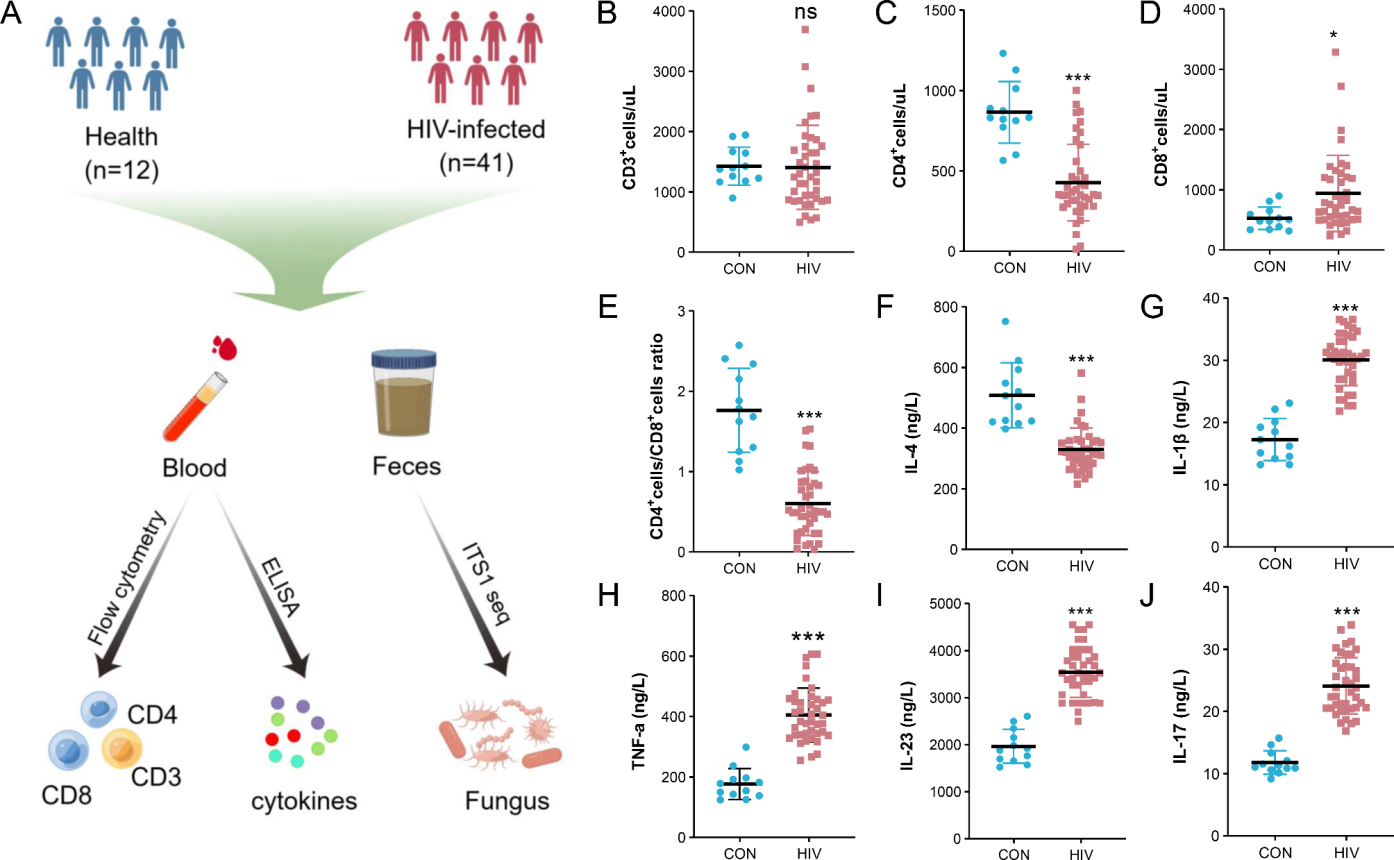
Technology roadmap and the levels of immune cells and inflammatory factors. **A** Technology roadmap. **B-E** The levels of immune cells. **F-J** The levels of inflammatory factors in the serum

Collecting fecal (about 5 g) and peripheral blood samples (about 5 mL) from PLWHA and healthy volunteers, and store them in -80 ℃. All study subjects did not use antibiotics within the past month before sampling. The Technology roadmap of this study is shown in Fig. 1A.

### Immune cells and serum cytokine detection

Flow cytometry was used to determine the levels of CD3^+^ T cells, CD4^+^ T cells, and CD8^+^ T cells in peripheral blood samples from PLWHA and healthy volunteers. The flow cytometer (Facscalibur, Becton Dickinson) was turned on and maintained normally, and quality control samples were tested on the flow cytometer. Extract 50–200 µL whole blood samples and perform cell lysis or separation and resuspension of mononuclear cells according to the experimental requirements. Add an appropriate amount of CD4/CD8/CD3 antibody (FITC/PE/Cy5) (Biorbet, #orb688641, UK) according to the color labeling scheme to label the corresponding fluorescence. Incubate at room temperature in the dark for 30 min. Flow cytometry was used for sample detection and analysis, and Cellquest software was used to obtain and analyze cells, with 10,000 cells obtained for each detection. Distinguish lymphocytes using scatter plots of forward scatter (FCS) and side scatter (SSC) scattered light, and use histograms to represent CD3, CD4, and CD8 positive lymphocytes, respectively.

The ELISA (Enzyme-linked Immunosorbent Assay) kit (purchased from Shanghai Meilian Biotechnology Co., Ltd., double antibody sandwich method) (R: 0.990, inter-assay CV%<10% and within-assay CV%<10%) was used to determine the levels of TNF-α, IL-1β, IL-4, IL-17 and IL-23 in serum according to the corresponding instructions.

### Intestinal fungal community detection

Fecal genome DNA extraction: 53 stool samples collected from PLWHA and health examinees were taken from the − 80 ℃ and thawed on ice, and then the total DNA of stool samples was extracted according to the operation instructions of Tiangen fecal DNA extraction kit. Nano Drop 2000 and agarose gel electrophoresis were used to detect the concentration and quality of DNA, and the qualified DNA was used for subsequent tests.

High-throughput sequencing of gut fungal communities: The ITS gene (The gene sequences of 18 S, 5.8 S, and 26 S in rDNA are highly conserved in eukaryotes, while ITS bears less selection pressure, has a relatively fast evolutionary speed, high variability, and is highly repetitive in the nuclear genome. Therefore, special primers can be designed based on the mutation sites in the conserved sequences for PCR amplification of the ITS region) was amplified using specific ITS primers, with forward primers ITS1 (5’-CTTGGTCATTTAGAGGAAGTAA-3’) and reverse primers ITS2 (5’- GCTGGTTCTTCATCGATGC-3’). Amplify qualified DNA through PCR. The amplification program is as follows: pre-denaturation at 98 ℃ for 5 min, followed by 25 cycles (denaturation at 98 ℃ for 30 s, annealing at 53 ℃ for 30 s, and extension at 72 ℃ for 45 s), extension at 72 ℃ for 10 min, and storage at 4 ℃. The PCR reaction system is: 5 × Trans Start Fast Pfu buffer 5 µL, 2.5 mmol/L d NTPs 2 µL, Forward primer (10 µMol/L) 1 µL, Reverse primer (10 µMol/L) 1 µL, Fast PFU DNA polymerase (5 U/µL) 0.25 µL, Template DNA 1 µL, ddH_2_O 14.75 µL. Evaluate the quality of the library on Qubit@2.0 Fluorometer (Thermo Scientific) and Agilent Bioanalyzer 2100 system. The qualified library is sequenced in high-throughput to obtain the original data file.

Bioinformatics analysis: The QIIME V.1.9.1 software package is used for quality filtering of sequences. Cluster at 99% similarity to obtain operational taxonomic units (OTUs) and remove chimeric sequences. Display the richness and diversity of fungal communities within the group through Alpha diversity analysis (Characterize richness with Chao1 and Observed species indexes, diversity with Shannon and Simpson indexes, uniformity with Pielou’s evenness index, and coverage with Good’s coverage index). Compare the fungal community composition between the two groups through beta diversity analysis (Principal co-ordinate analysis, PCoA), and display the differences between the groups. The Unite ITS database was used to classify the fungi. T-test analysis and comparison of fungal species composition and differences between the two groups. LEfSe analysis was used to identify fungal species with statistically significant differences between groups.

### Phylogenetic investigation of communities by reconstruction of unobserved states (PICRUSt2) analysis

PICRUSt2 can predict the function of ITS gene sequences in the MetaCyc database. Firstly, align the ITS gene sequences of known microbial genomes, construct an evolutionary tree, and infer the gene function spectrum of their common ancestors [2]. Align the ITS feature sequence with the reference sequence to construct a new evolutionary tree [3]. Using the Castor hidden state prediction algorithm, based on the gene family copy number corresponding to the reference sequence in the evolutionary tree, infer the nearest sequence species of the feature sequence, and then obtain its gene family copy number [4]. Calculate the copy number of gene families for each sample based on the abundance of feature sequences [5]. Finally, the gene family is mapped to various databases, and MinPath is used by default to infer the existence of metabolic pathways, thereby obtaining abundance data of metabolic pathways in each sample.

### Correlation analysis between intestinal fungal community and serum cytokines

In order to analyze the correlation between microbiota and cytokines (Pearson correlation), Using OmicShare Tools (https://www.OmicShare.com/Tools) to analyze the correlation between the top 10 genera and cytokines (CD3^+^, CD4^+^, CD8^+^, CD4^+^/CD8^+^, TNF-α, IL-1β, IL-4, IL-17 and IL-23).

### Statistical analysis

SPSS 24.0 software was used to compare and statistically analyze data, and the differences between the two groups were analyzed using the Student *t* test. Inspection level α = 0.05. All data are expressed as mean ± standard deviation ($\stackrel{-}{\text{x}}$ ± s).

## Results

### Immune cells and inflammatory factor levels

There was no statistically significant difference in the number of CD3^+^ T cells between healthy volunteers and PLWHA (*P* > 0.05). However, the ratio of CD4^+^/CD8^+^ T cells in the peripheral blood of PLWHA was significantly lower than that of healthy volunteers (*P* < 0.001). In addition, the levels of pro-inflammatory factors (IL-1β, IL-17, IL-23 and TNF-α) in the peripheral blood of PLWHA were significantly higher than those of healthy volunteers (*P* < 0.001), while their anti-inflammatory factor levels (IL-4) were significantly lower than those of healthy volunteers (*P* < 0.001) (Fig. 1B-J).

### Diversity of intestinal fungal community

**Fig. 2:**
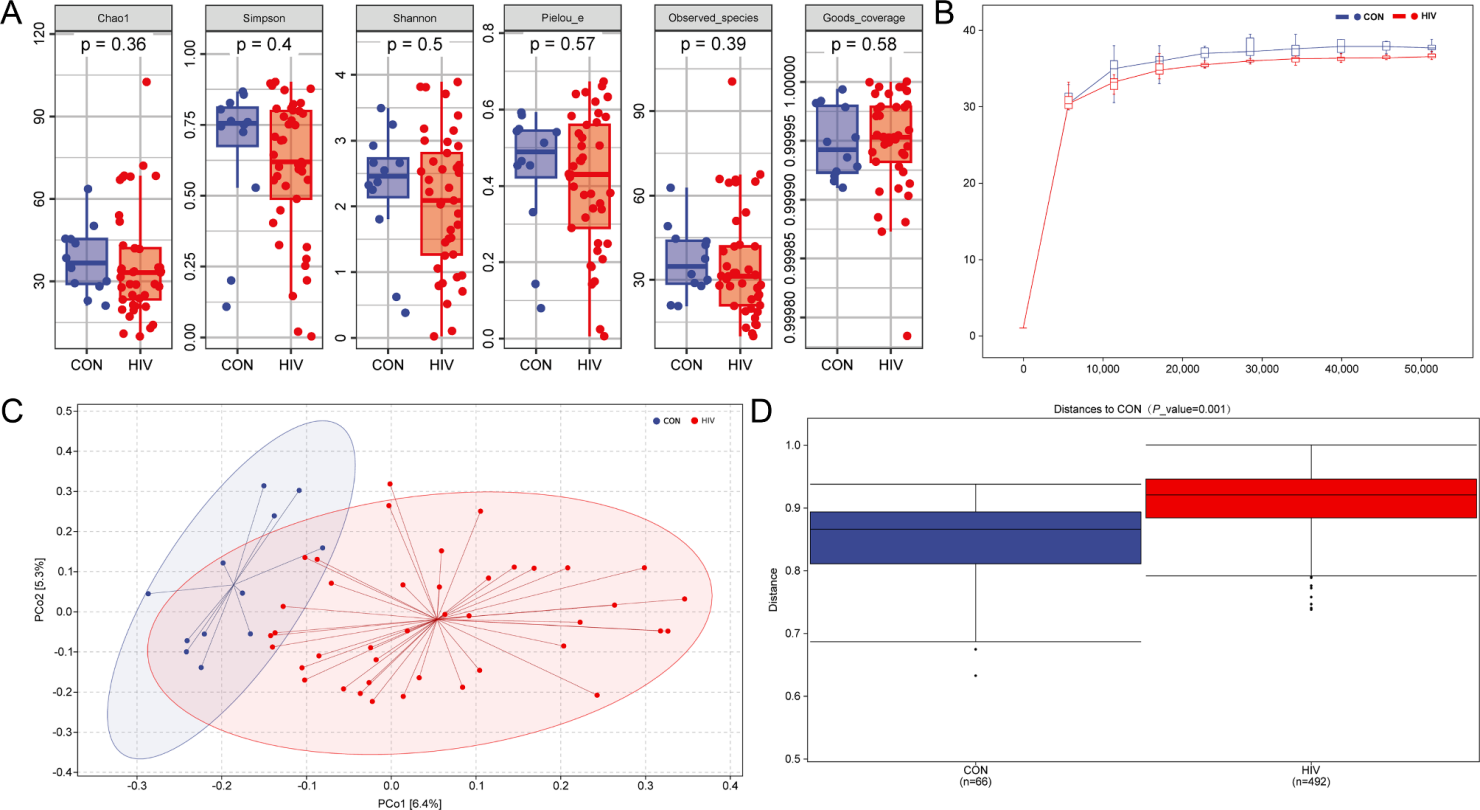
Alpha and beta diversity of intestinal fungal community. **A** Alpha diversity of intestinal fungal community. **B** Sparse curve. **C** PCoA analysis. **D** Analysis of differences between Con and HIV groups

There was no significant difference in Alpha diversity between PLWHA and healthy volunteers (Chao1, Simpson, Shannon, Pielou_e, Observed species and Good’s coverage indexes) (*P* > 0.05) (Fig. 2A). The sparse curve results based on the Chao1 index show that the current sequencing results are sufficient to reflect the diversity contained in the current sample, and further increasing the sequencing depth cannot detect a large number of undiscovered operational taxonomic units OTUs (Fig. 2B). The results of PCoA showed that the dispersion degree among the samples in the PLWHA group was relatively large, and there was a significant difference between the PLWHA group and the healthy volunteer group (*P* < 0.01) (Fig. 2C). The results of group difference analysis showed that the distance between PLWHA and healthy volunteers was significantly different (*P* < 0.01) (Fig. 2D).

### Composition of intestinal fungal microbiota

**Fig. 3:**
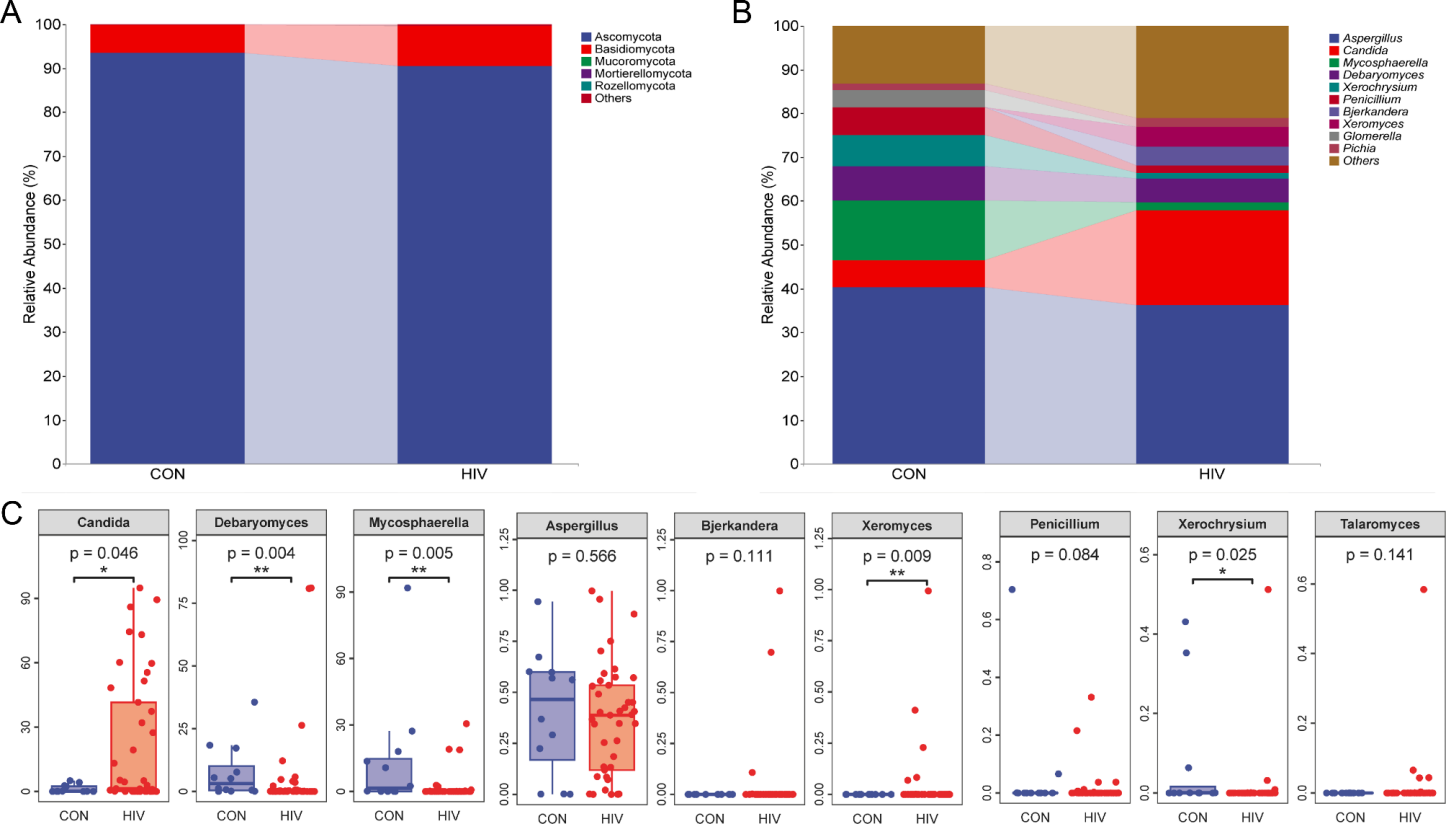
Composition of intestinal fungal community. **A** phylum level. **B** Genus level. **C** The relative abundance of *Candida*, *Debaryomyces* and *Mycosphaeralla*

At the phylum level, the relative abundance of Ascomycota in PLWHA is significantly lower compared to healthy volunteers (*P* < 0.01), whereas the relative abundance of Basidiomycota is significantly higher in PLWHA compared to healthy volunteers (*P* < 0.01) (Fig. 3A). At the genus level, the relative abundance of *Candida* and *Xeromyces* in PLWHA was significantly higher than that of healthy volunteers (*P* < 0.01), while the relative abundance of *Debaryomyces, Mycosphaerella* and *Xeroxysium*in PLWHA was significantly lower than that of healthy volunteers (*P* < 0.01 or *P* < 0.05) (Fig. 3B&C). Under physiological conditions, the dominant fungus *Candida* in the human digestive tract is in a symbiotic state with the human body. Under pathological conditions, *Candida* can induce direct damage to epithelial cells by adhering to epithelial cells and inducing the production of inflammatory mediators. This result suggests that some digestive symptoms in PLWHA not only require attention to changes in bacterial communities, but also to changes in fungal communities (Especially *Candida*).

### Analysis of species differences and marker species

**Fig. 4:**
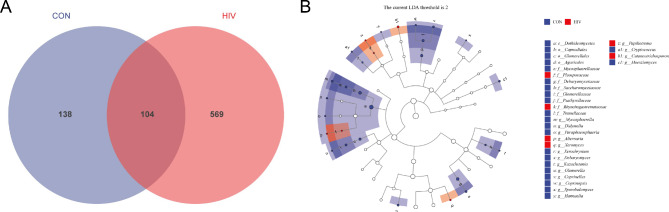
Analysis of species differences and marker species. **A** Venn Diagram. **B** LEfSe analysis. Note: The taxonomic branch map shows the taxonomic hierarchy of the main taxa from phylum to genus (from inner circle to outer circle) in the sample community. The node size corresponds to the average relative abundance of the classification unit; Hollow nodes represent classification units with insignificant inter group differences, while nodes with other colors (such as green and red) indicate that these classification units exhibit significant inter group differences and are more abundant in the group samples represented by that color. The letters indicate the names of taxonomic units with significant differences between groups

After exploring the differences in microbial community composition (i.e. beta diversity), we also need to know which species’ distribution differences are mainly caused by these differences (Venn and LEfSe analysis). Using Venn diagram to count the number of members of PLWHA and healthy individuals, which is unique to each group and the number of OTUs shared between groups. Using LEfSe analysis can directly perform differential analysis at all classification levels simultaneously, and at the same time, LEfSe emphasizes finding robust differential species between groups, namely biomarkers. One of its major characteristics is that it is not only limited to analyzing the differences in community composition among different sample groups, but can also delve into different subgroups to select the marker microbial groups that perform consistently in different subgroups. The Venn diagram results show that PLWHA and healthy volunteers have 569 and 138 unique OUTs, respectively, with 104 shared OUTs between the two groups (Fig. 4A). The LEfSe analysis results reveal that at the family level, Pleosporaceae and Rhynchogastremataceae are dominant fungal families in PLWHA, with their relative abundance significantly higher compared to healthy volunteers (*P* < 0.01). At the genus level, *Alternaria*, *Xeromyces*, *Papiliotrema* and *Cutaneotrichosporon* are dominant fungal genera in PLWHA, with their relative abundance significantly higher compared to healthy volunteers (*P* < 0.01) (Fig. 4B).

### PICRUSt2 functional potential prediction

**Fig. 5:**
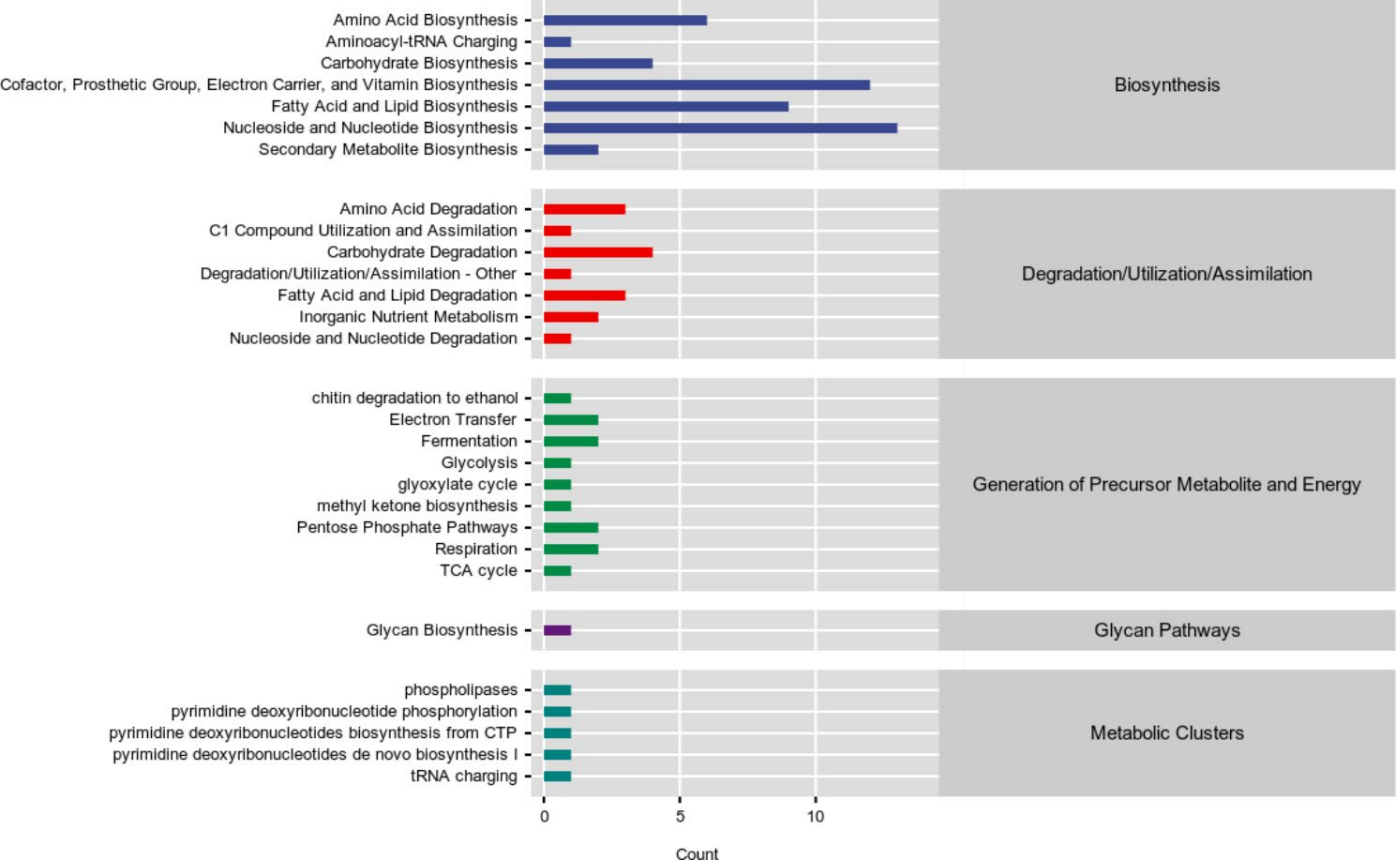
Statistical analysis of metabolic pathways (MetaCycle)

MetaCycle is the largest metabolic reference database in the field of life sciences that has been elucidated through experimental data. MetaCyc contains various pathways involved in primary and secondary metabolism, as well as related metabolites, biochemical reactions, enzymes, and genes. It aims to classify all metabolic processes in life by storing representative experimentally validated metabolic pathways. The results of this study show that there were significant differences in the metabolic pathways of biosynthesis (nucleoside and nucleotide biosynthesis, cofactor, prosthetic group, electron carrier, and vitamin biosynthesis) between the gut fungal communities of PLWHA and healthy individuals (Fig. 5).

### Correlation analysis between intestinal fungal community and serum cytokines

**Fig. 6:**
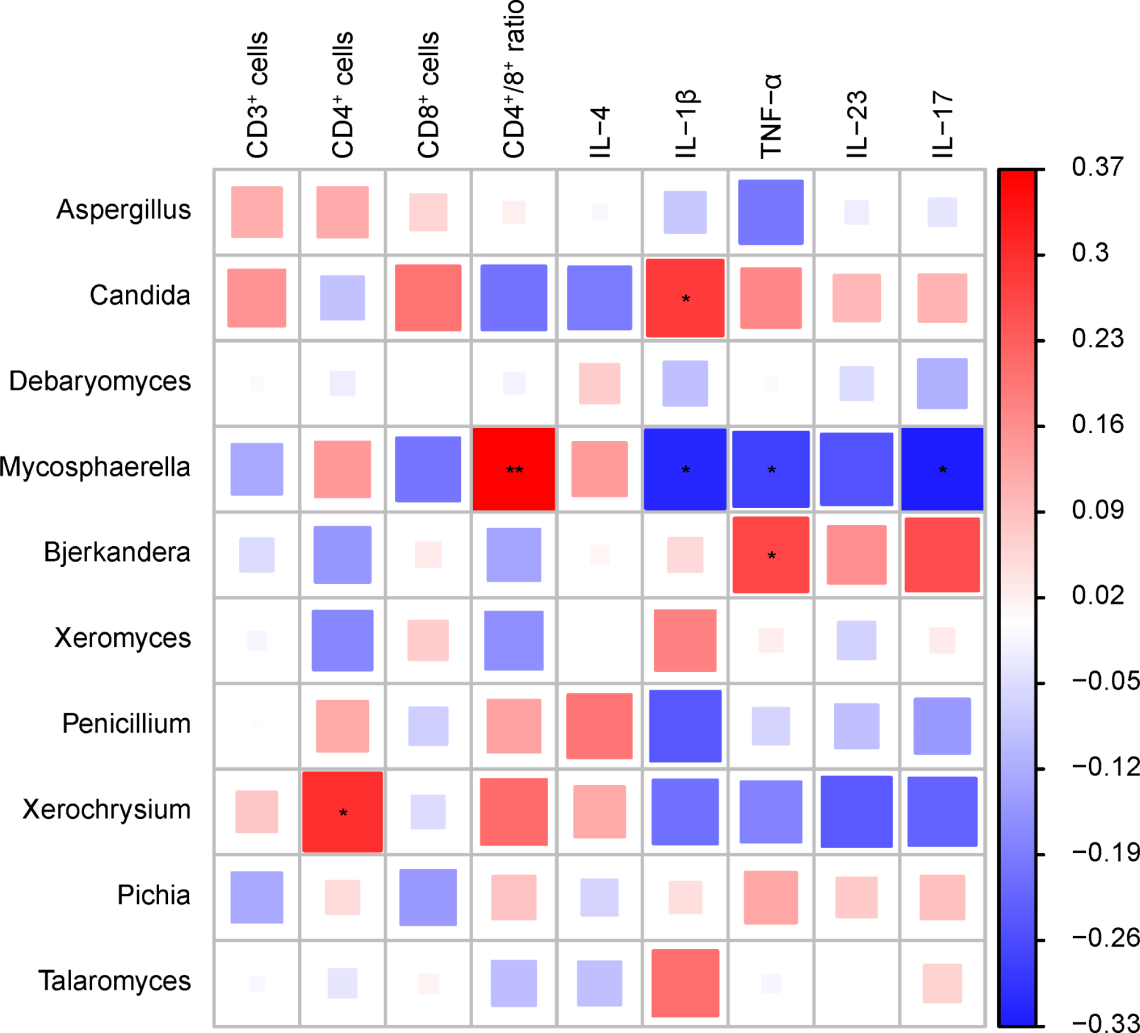
Correlation analysis between differential fungal genera and immune cells, inflammatory factors

The correlation analysis results show that *Mycospaerella* and *Xeromyces* are significantly positively correlated with CD4^+^/CD8^+^ T cells and the anti-inflammatory cytokine IL-4, while they are significantly negatively correlated with pro-inflammatory cytokines (IL-1β, IL-17, IL-23 and TNF-α). On the other hand, *Candida* is negatively correlated with CD4^+^/CD8^+^ T cells and the anti-inflammatory cytokine IL-4, while it is positively correlated with pro-inflammatory cytokines (IL-1β, IL-17, IL-23, and TNF-α). (Fig. 6)

## Discussion

The role of gut microbiota in the gut homeostasis of PLWHA is receiving increasing attention [27]. Previous studies have primarily focused on determining the bacterial diversity in the gut of PLWHA [28–30]. Although fungal communities account for less than 1% of the human microbiota, they play a crucial role in maintaining the balance of the microbial community, stable metabolic functions, and host immune responses [31]. This study compared the differences in gut fungal communities and cytokines between healthy individuals and PLWHA.

The study found significant changes in gut fungal communities in PLWHA, with a significant increase in the relative abundance of *Candida*, *Bjerkandera*, and *Xeromyces*, while the relative abundance of *Mycospaerella*, *Xeroxysium*, *Penicillium*, and *Glomerella* significantly decreased. It is suggested that *Candida*, *Bjerkandera*, and *Xeromyces* may be key factors in further disrupting the microbial ecology. *Candida* is an opportunistic pathogen in humans, and its pathogenicity is associated with compromised host immune systems [32]. The formation of hyphae, phenotypic switching, and secretion of proteases contribute to its virulence, with 10 SAP genes encoding secretory aspartic proteases being key to its pathogenicity [33–35]. After *Candida* infection, it is attacked by the host’s innate immune system, which degrades host complement components by secreting proteins such as Sap1, Sap2, and Sap3 to evade the host’s immune defenses [36]. Sap1 also plays an important role in *Candida* albicans mucosal colonization, increasing adhesion intracellularly and causing tissue infiltration extracellularly [33]. In addition, *Candida* utilizes the intestinal immune damage caused by HIV and the dysbiosis of bacterial flora in the intestinal environment to promote its own growth and high expression of toxic protein SAP1 [37]. The abundance of *Candida* in the intestines of PLWHA significantly increases, and the structure of gut fungal community changes [38]. Overexpression of the toxic gene SAP1 related to *Candida albicans* may enhance its ability to adsorb intestinal mucosa and colonize in vivo [39]. Meanwhile, SAP1 can further exacerbate mucosal barrier damage in PLWHA by degrading host complement factors and disrupting intestinal immunity [35, 40]. Developing precise interventions targeting the microbiota, correcting fungal dysbiosis in the gut by introducing exogenous microorganisms, and/or inhibiting SAP1 activity, may reduce damage to the intestinal mucosal barrier and delay disease progression [41]. In summary, Sap1 mediates multiple processes in *Candida albicans*, including adhesion to host mucosal epithelial cells, lysis of host cells, and immune evasion [42].

*Debaryomyces* may be a potential probiotic. The research results of Guo et al. showed that *Debaryomyces* has a good effect on antibiotic-related diarrhea. It can promote the growth of intestinal *Bifidobacteria* and *Lactobacilli* in mice and regulate the balance of intestinal microecology [43]. Another potential medical application of *Debaryomyces* involves their ability to express antipathogenic proteins known as killer toxins or mycocins of pathogenic yeasts (e.g. *Candida*), Banjara et al’s studies in vitro experimental have shown that *Debaryomyces* can produce killing toxins that inhibit the growth of *Candida albicans* and *Candida tropicalis* [41]. In this study, the abundance of *Debaryomyces* in PLWHA group was significantly reduced. This may be due to the drastic changes in the intestinal immune environment caused by HIV infection, leading to a significant reduction in the colonization of *Debaryomyces*. On the one hand, it may lead to its weakened control of *Candida*. On the other hand, these beneficial fungi may also be affected in synthetic metabolism of short-chain fatty acids and vitamins. Thereby affecting the intestinal health of the host.

The immune dysregulation resulting in microbial imbalance depends on the early study of imbalance [44]. This study found a correlation between the immune levels and gut fungal communities in PLWHA, with *Candida* exhibiting a positive correlation with inflammatory factors and a negative correlation with anti-inflammatory factors and CD4^+^/CD8^+^ T cells. Previous studies have found that HIV infection significantly damages intestinal immune cells, and as CD4^+^ T lymphocyte counts decrease, immune function is significantly impaired, leading to microbial dysbiosis, overgrowth of pathogenic bacteria in the intestines, immune escape, and accelerated intestinal smooth muscle contractions, leading to the occurrence of symptoms such as diarrhea [45]. HIV-associated diarrhea includes infectious diarrhea (protozoa, fungi, viruses, bacteria, etc.) and non-infectious diarrhea (intestinal diseases, fat absorption disorders, antiviral drugs, tumors), with gastrointestinal immune dysfunction and intestinal dysbiosis considered the two main causes of HIV-related diarrhea, with intestinal microbial infections in PLWHA occurring 20–100 times more frequently than in the general population [46, 47].

This study found that PLWHA have altered gut fungal profiles and immune functions. The increased relative abundance of *Candida* may be one of the potential factors triggering microbial dysbiosis and intestinal mucosal damage in PLWHA, and a risk factor for substantial intestinal mucosal damage leading to the onset of AIDS. The results of this study provide additional information on the structural characteristics of fungal communities in the gut microbiota of PLWHA and their correlation with gut immune function. In the future clinical treatment of AIDS-related digestive tract diseases, in addition to paying attention to bacterial communities, we should also pay attention to the impact of fungal communities, especially *Candida*, on the host intestinal immune microenvironment. For the subsequent development of precise interventions targeting intestinal fungal community disorders, including correcting intestinal fungal community disorders by introducing exogenous microorganisms, or inhibiting the damage of related fungal to the intestinal mucosal barrier, providing a basis for improving gastrointestinal symptoms in PLWHA. In addition, it should be noted that the small sample size and insufficient research depth are the limitations of this study, and it is necessary to expand the sample size and attempt to correct the disordered fungal community in future studies.

## Conclusions

The fungal diversity in the gut of PLWHA undergoes significant changes, with a significant increase in the relative abundance of *Candida*, *Bjerkandera*, and *Xeromyces and* a significant decrease in the relative abundance of *Mycospaerella*, *Xeroxysium*, *Penicillium* and *Glomerella* being the main characteristics. These changes are accompanied by a significant decrease in the CD4^+^/CD8^+^ T cell ratio and the anti-inflammatory factor IL-4, as well as a significant increase in pro-inflammatory factors (IL-1β, IL-17, IL-23 and TNF-α).

### Electronic supplementary material

Below is the link to the electronic supplementary material.

**Supplementary Material 1: S1.** Inclusion and exclusion criteria.

## Data Availability

The ITS gene sequencing datasets generated and/or analyzed during the present study are available at NCBI project PRJNA994610.

## Abbreviations

AIDS: Acquired immune deficiency syndrome
Internal Transcribed Spacer: ITS
*Escherichia coli*: *E. coli*
Interleukin: IL
Tumor necrosis factor: TNF
SCFAs: Short chain fatty acids
PCoA: Principal Co-ordinates Analysis
