# Supplementary material for: Characterization of intestinal fungal community diversity in people living with HIV/AIDS (PLWHA)
Source: AIDS Res Ther. 2024 Feb 13;21:10. doi: 10.1186/s12981-023-00589-x (PMC10863270; doi:10.1186/s12981-023-00589-x)
Supplement: Supplementary file 1 — Supplementary Material 1: S1. Inclusion and exclusion criteria. [file 12981_2023_589_MOESM1_ESM.docx]

1. Inclusion criteria

(1) HIV antibody is positive and confirmed to meet the diagnostic criteria for AIDS virus infection by diagnostic tests;

(2) CD4+T cells>200/ μ L; The HIV virus load in the treatment group was less than 40 copies/ml;

(3) Age 18-60 years old, with normal body mass index;

(4) No symptoms of chronic infections such as fatigue, hot flashes, night sweats, etc. within at least one month, no significant changes in weight, and no use of antibiotics, probiotics, or other preparations in the past month.

(5) Expected survival time>2 years;

(6) Volunteer to join this study and sign an informed consent form;

(7) No other sexually transmitted diseases.

2. exclusion criteria

(1) Those who have not undergone confirmed experiments and meet the diagnostic criteria for HIV infection;

(2) Those who do not understand the purpose of this study and are unwilling to sign an informed consent form;

(3) Those who have taken antibiotics and have an irregular diet within the past month;

(4) Individuals with multiple organ damage.
